# Dataset on technological alliances by using joint patents in the biotechnology industry

**DOI:** 10.1016/j.dib.2020.106124

**Published:** 2020-08-04

**Authors:** Hugo E. Martínez Ardila, Luis C. Gómez Flórez, Juan F. Guarín Castro

**Affiliations:** a Escuela de Estudios Industriales y Empresariales, Universidad Industrial de Santander, Cra 27, Calle 9, La Universidad, Bucaramanga, Colombia; b Universidad Manuela Beltrán, Colombia

**Keywords:** Biotechnology, Joint patents, Innovation value, Technological alliances

## Abstract

The data in this article includes biotechnological joint patents as a proxy of technological alliances. Joint patents are not directly found in public or commercial databases. Each joint patent has their corresponding International Patent Classification codes, their forward citation measure as an indicator of innovation value, and the firms involved in the process. The data was collected during a time frame that ranges from 2006 to 2010. Data from the joint biotechnological patents was collected at first during the time window, and then filtered, cleaned, and sampled accordingly to obtain 340 joint patents between firms (e.g. alliances) in the industry. Then each of the joint patents was processed to find the forward citations they have. Data on joint patents of biotechnology industry can be reused in future studies related to technological alliances and patenting mechanisms and process.

 **Specifications Table**

**Table:** 

**Subject**	Management of Technology and Innovation
**Specific subject area**	Technological alliances, innovation value.
**Type of data**	Tables
**How data were acquired**	First, data were collected from the USPTO database using the specialized software Matheo Patent XE® and then filtered, sampled and completes by the researchers.
**Data format**	Raw Filtered
**Parameters for data collection**	Data was extracted from the United States Patent and Trade Organization (USPTO) during the years 2006 to 2010. The sample consists of 340 joint patents from the biotechnology industry, including the number of forward citations to each joint patent in a time window of five years.
**Description of data collection**	Firstly, it was necessary to obtain all the biotechnology patents in the United States Patent and Trade Organization (USPTO) during the years 2006–2010. The second step consisted on extract the joint biotechnology patents from this database by checking and processing the number of patent applicants. Thirdly, a random sample method was used to select a subset in order to estimate characteristics of the whole population. This sample was rechecked and cleaned to improve the quality of data. Finally, a search and processing of forward citations received in subsequent patents in a time window of five years was made to each of the joint patents.
**Data source location**	Institution: United States Patent and Trade Organization (USPTO)Country: United States of America
**Data accessibility**	Repository name: MendeleyDirect URL to data: https://data.mendeley.com/datasets/jcpj3xdhtt/draft?a=bafa219f-f1f1-4fe5-a459-2a2672c47930

## Value of the data

• The dataset provides information about joint patents which are not directly found in databases, specifically in the biotechnology sector. The data set can be used as a reference to the improve understanding about technological alliances or R&D collaboration and how partner technological attributes influence the value of the joint innovations.
• Patent offices or patent organizations, firms from the biotechnology sector that have the will to develop potential strategic alliances, an managers and researchers will benefit from this data related to joint patents as a measure of firms collaborating in technological alliances.
• Data can be used as a reference or baseline to develop comparative studies. Comparative studies should include analysis in terms of the value of the joint patent by means other than forward citations (e.g. economic value, private value); also, can be used as a reference to other databases different from the USPTO, such as the European Patent Office, or the Japan Patent Office to find differences and similitudes on the mechanisms used on those regions.

## 1 Data description

The dataset sample is composed of joint patents in the biotechnology industry. Patents in biotechnology industry are an indication of innovative success and the generation of new knowledge [1, 2]. The biotechnology joint patents in the data set are those that have the biotechnology classes according to the International Patent Classification -IPC- [3].

Each joint patent or co-patent shown in the dataset is defined as the patent owned by two or more firms [4] that form a duopoly or tight oligopoly comparable to a restrictive licensing agreement from an economic standpoint [5]. The joint patents in the dataset are considered a good measure of innovative output in an alliance [6, 7]. Following past research, the patents used in the dataset are those that have been successfully applied for [8].

In general, there is a distinction between the private value and the social value of inventions or patents [9]. The private value are the benefits perceived by the firm that has the patent compared to the firm that does not have it [10]. The social value embodies the total net value generated by the patent for social welfare [9]. The present dataset make use of one of the most used indicators of patent social value, the number of forward citations [11]. This indicator has been used already in valuing joint patents [4,7,12].

The dataset contains a sample of 340 joint patents (technological alliances) in the biotechnology industry with a total and average forward citation of 570 and 1.67 respectively, in a five-year window; the maximal forward citation made to a joint patent is 28 and the minimal forward citation for each year is cero. Table 1 shows a basic description of the aggregate data obtained per year.

**Table 1:** Aggregate information of joint patents data set by year from 2006 to 2010

Year	# of joint patents	Total Forward citations	Mean Forward citations	Max Forward Citation
2006	15	26	1.73	7
2007	67	174	2.59	28
2008	94	159	1.69	21
2009	89	151	1.69	26
2010	75	60	0.8	7

**Fig. 1:**
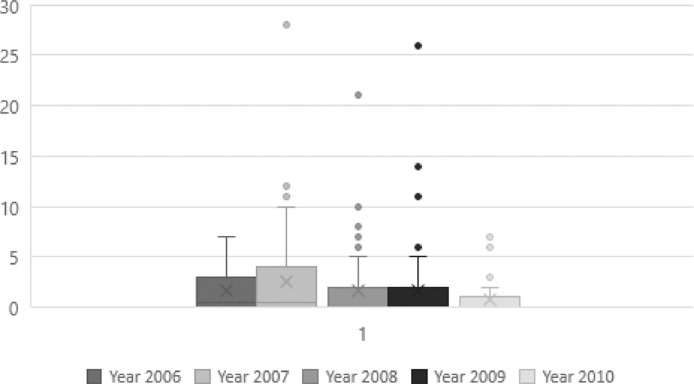
Boxplot of forward citation for each year.

Fig. 1 shows the boxplot by year of the forward citation variable. In all the years, the forward citations received by the joint patents are more disperse between the 50% (Q2) and the 75% (Q3) of the dataset. There is always a concentration in the first 25% of the data. According to this figure, the mean is always bigger to the median in all the years. The year 2007 offers the bigger dispersion and according to Table 1 is the year with the bigger forward citation indicator. However, 2008 is the year with the higher number of joint patents. The years 2007, 2008, 2009, and 2010 have outliers.

**Fig. 2:**
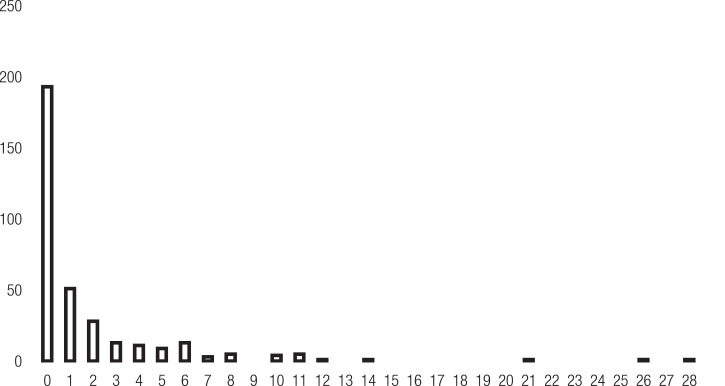
forward citation (joint value) frequency distribution.

Fig. 2 shows the forward citation distribution of the joint patents. Most of the joint patents from the sample (56%) are not cited at all, 15% are cited once, 8% are cited twice, and 3% are cited three times, and only 4% of the joint patents are cited equall or greater than 10 times. This figure shows how skewed is the distribution of the count dependent variable.

The Appendix A shows the complete dataset that includes the year of application, the application number, the 5-year forward citation, the International Patent Classification codes for the biotechnology industry, and the pair of firms that are assignees of each one of the joint patents. Appendix B shows the complete raw data from which the dataset was sampled.

## 2 Experimental design, materials, and methods

The dataset is obtained from the joint patents in the biotechnological industry. The time frame used ranges from the years 2006 to 2010. Data were obtained from joint technological patents according to the International Patent Classification [3]. The technological codes or classes used to filter the patents are: A01H1/00, A01H4/00, A61K38/00, A61K39/00, A61K48/00, C02F3/34, C07G(11/00, 13/00,15/00), C07K(4/00,14/00,16/00,17/00,19/00), C12M, C12N, C12P, C12Q, C12S, G01N27/327, G01N33/(53*,54*,55*,57*,68,74,76,78,88,92). Table 2 offers a description of the IPC codes used to obtain the dataset for the biotechnology industry.

**Table 2:** International patent classification code description for biotechnology patents [3]

IPC codes	Description
A01H 1/00	Processes for modifying genotypes
A01H 4/00	Plant reproduction by tissue culture techniques
A61K 38/00	Medicinal preparations containing peptides
A61K 39/00	Medicinal preparations containing antigens or antibodies
A61K 48/00	Medicinal preparations containing genetic material which is inserted into cells of the living body to treat genetic diseases; Gene therapy
C02F 3/34	Biological treatment of water, waste water, or sewage: characterized by the microorganisms used
C07G 11/00	Compounds of unknown constitution: antibiotics
C07G 13/00	Compounds of unknown constitution: vitamins
C07G 15/00	Compounds of unknown constitution: hormones
C07K 4/00	Peptides having up to 20 amino acids in an undefined or only partially defined sequence; Derivatives thereof
C07K 14/00	Peptides having more than 20 amino acids; Gastrins; Somatostatins; Melanotropins; Derivatives thereof
C07K 16/00	Immunoglobulins, e.g. monoclonal or polyclonal antibodies
C07K 17/00	Carrier-bound or immobilized peptides; Preparation thereof
C07K 19/00	Hybrid peptides
C12M	Apparatus for enzymology or microbiology
C12N	Micro-organisms or enzymes; compositions thereof
C12P	Fermentation or enzyme-using processes to synthesize a desired chemical compound or composition or to separate optical isomers from a racemic mixture
C12Q	Measuring or testing processes involving enzymes or micro-organisms; compositions or test papers therefor; processes of preparing such compositions; condition-responsive control in microbiological or enzymological processes
C12S	Processes using enzymes or micro-organisms to liberate, separate or purify a pre-existing compound or composition processes using enzymes or micro-organisms to treat textiles or to clean solid surfaces of materials
G01N 27/327	Investigating or analysing materials by the use of electric, electro-chemical, or magnetic means: biochemical electrodes
G01N 33/53*	Investigating or analysing materials by specific methods not covered by the preceding groups: immunoassay; biospecific binding assay; materials therefore
G01N 33/54*	Investigating or analysing materials by specific methods not covered by the preceding groups: double or second antibody: with steric inhibition or signal modification: with an insoluble carrier for immobilizing immunochemicals: the carrier being organic: synthetic resin: as water suspendable particles: with antigen or antibody attached to the carrier via a bridging agent: Carbohydrates: with antigen or antibody entrapped within the carrier.
G01N 33/55*	Investigating or analysing materials by specific methods not covered by the preceding groups: the carrier being inorganic: Glass or silica: Metal or metal coated: the carrier being a biological cell or cell fragment: Red blood cell: Fixed or stabilized red blood cell: using kinetic measurement: using diffusion or migration of antigen or antibody: through a gel
G01N 33/57*	Investigating or analysing materials by specific methods not covered by the preceding groups: for venereal disease: for enzymes or isoenzymes: for cancer: for hepatitis: involving monoclonal antibodies: involving limulus lysate
G01N 33/68	Investigating or analysing materials by specific methods not covered by the preceding groups: involving proteins, peptides or amino acids
G01N 33/74	Investigating or analysing materials by specific methods not covered by the preceding groups: involving hormones
G01N 33/76	Investigating or analysing materials by specific methods not covered by the preceding groups: human chorionic gonadotropin
G01N 33/78	Investigating or analysing materials by specific methods not covered by the preceding groups: thyroid gland hormones
G01N 33/88	Investigating or analysing materials by specific methods not covered by the preceding groups: involving prostaglandins
G01N 33/92	Investigating or analysing materials by specific methods not covered by the preceding groups: involving lipids, e.g. cholesterol

The data was first filtered by finding the joint patent applications found in the United States Patent and Trade Organization – USPTO- database. The USPTO has information on patent applications and all classes of patents such as utility, design, reissue, plant patents, and includes bibliographic data, full description of the invention and the claims. The USPTO was chosen because the United States is the country with the higher share in biotechnology patents filed under the Patent Cooperation Treaty -PCT_ with a 40.94% [13]. Other databases like the European Patent Office were not mixed in the dataset, because the practices about patent processing are different between offices [14].

There is not direct way to obtain biotechnology joint patents from patent databases. To do so, the first step in this process was to obtain all the biotechnology patents no matter how many owners were assigned to them. This activity was made using the standalone software Matheo Patent XE® by means of a search equation based on the IPC codes assigned to the field. Matheo Patent XE® is a tool that search, analyses and make survey of patents. The search equation used in the analysis was the following:

((ic:(A01H1) OR ic:(A01H4) OR ic:(A61K38) OR ic:(A61K39) OR ic:(A61K48) OR ic:(C02F3/34) ORic:(C07G11) OR ic:(C07G13) OR ic:(C07G15) OR ic:(C07K4) OR ic:(C07K14) OR ic:(C07K16) ORic:(C07K17) OR ic:(C07K19) OR ic:(C12M*) OR ic:(C12N*) OR ic:(C12P*) OR ic:(C12Q*) OR ic:(C12S*)OR ic:(G01N27/327) OR ic:(G01N33/53) OR ic:(G01N33/531) OR ic:(G01N33/532) OR ic:(G01N33/533)OR ic:(G01N33/534) OR ic:(G01N33/535) OR ic:(G01N33/536) OR ic:(G01N33/537) OR ic:(G01N33/538)OR ic:(G01N33/539) OR ic:(G01N33/54) OR ic:(G01N33/541) OR ic:(G01N33/542) OR ic:(G01N33/543)OR ic:(G01N33/544) OR ic:(G01N33/545) OR ic:(G01N33/546) OR ic:(G01N33/547) OR ic:(G01N33/548)OR ic:(G01N33/549) OR ic:(G01N33/55) OR ic:(G01N33/551) OR ic:(G01N33/552) OR ic:(G01N33/553)OR ic:(G01N33/554) OR ic:(G01N33/555) OR ic:(G01N33/556) OR ic:(G01N33/557) OR ic:(G01N33/558)OR ic:(G01N33/559) OR ic:(G01N33/57) OR ic:(G01N33/571) OR ic:(G01N33/572) OR ic:(G01N33/573)OR ic:(G01N33/574) OR ic:(G01N33/575) OR ic:(G01N33/576) OR ic:(G01N33/577) OR ic:(G01N33/578)OR ic:(G01N33/579) OR ic:(G01N33/68) OR ic:(G01N33/74) OR ic:(G01N33/76) OR ic:(G01N33/78) ORic:(G01N33/88) OR ic:(G01N33/92))

In order to filter actors in the patents different from firms, the “inventor” filter was used, followed by the filter equation:

not APP/univ* and not APP/empty and not APP/colle* and not APP/inst* and not APP/center and not APP/hospital*

The results from the software offered data that included joint and single owner patents. Therefore, the second step consisted on extracting only the joint biotechnological patents from this initial data. To do so, was necessary to filter the patents that had more than one applicant in the applicant field, this was done by the authors using a spreadsheet of Microsoft Excel®. The total number of joint patents in the biotechnological field was 1931 (Appendix B). This set of joint patents is the universe of the research. A simple random sampling method was used to select a subset to estimate characteristics of the whole population. The resulting sample size was 465 joint patents.

The 465 joint patents were cleaned with high detail in order to overcome problems like incomplete or confused information or when they belonged to organizations or actors different from firms. The resulting sample of joint patents after cleaning was 340 patents which is the dataset used (Appendix A). In order to obtain a 5% of margin error, 95% of confidence level and a response distribution of 50%, the sample of joint patents would have been of 320. Once the 340 biotechnological joint patents were obtained, was necessary to find the forward citation to each one of them; in other words, the number of citations received by each joint patent application in the next five years from the application date. Consequently, the forward citations were collected from the years 2006 to 2014.

## Declaration of Competing Interest

The authors declare that they have no known competing financial interests or personal relationships which have, or could be perceived to have, influenced the work reported in this article.
